# Aerosol-assisted CVD of thioether-functionalised indium aminoalkoxides

**DOI:** 10.1007/s00706-017-1980-2

**Published:** 2017-06-02

**Authors:** Felix Biegger, Felix Jungwirth, Michael Stanislaus Seifner, Christoph Rameshan, Sven Barth

**Affiliations:** Institute of Materials Chemistry, TU Wien, Getreidemarkt 9, Vienna, 1060 Austria

**Keywords:** AACVD, Oxysulphides, Indium aminoalcoholates, Thin films, Nanowires

## Abstract

**Abstract:**

Thioether-functionalised indium aminoalcoholates have been used as single-source precursors in aerosol-assisted CVD processes. The obtained In_2_O_3−*x*_S_*x*_ oxysulphide deposits show either a single indium sulphide phase for deposits with high sulphide content [>75% (S/(S + O)) for the *t*-butyl derivatives] or pronounced phase separation in indium oxide and indium sulphide for lower sulphide content [<62% (S/(S + O)) for the *n*-butyl derivatives]. In addition to thin films, polycrystalline 1D structures are obtained at slightly modified synthesis conditions. The materials are analysed by EDX, XRD, XPS, SEM, and TEM.

**Graphical abstract:**

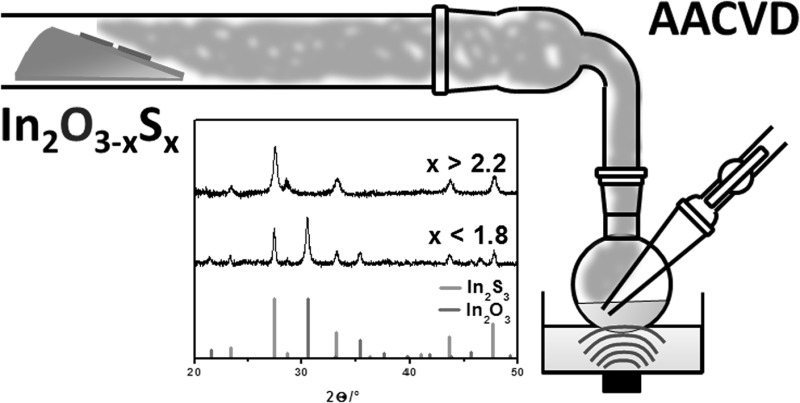

**Electronic supplementary material:**

The online version of this article (doi:10.1007/s00706-017-1980-2) contains supplementary material, which is available to authorized users.

## Introduction

Indium oxide In_2_O_3_ is a wide band gap material with a direct band gap of 2.9–3.0 eV and transparency in the visible range of the spectrum [1]. Therefore, In_2_O_3_ has been widely used in optoelectronics, sensing and as a transparent conducting oxide [2, 3]. Similarly, indium sulphide In_2_S_3_ with a band gap of approximately 2.2 eV has been largely exploited for optoelectronics and as buffer layer in photovoltaic devices [4, 5].

In contrast, reports on indium oxysulphides are rare despite the fact that they could be a favourable replacement for CdS in buffer layers of Cu(In,Ga)Se solar cells [6]. The term oxysulphide represents in this paper a material composition with oxide and sulphide ions In_2_O_3−*x*_S_*x*_, which can be related to a sulphide phase with substitutional oxygen in In_2_S_3_ or phase-separated In_2_O_3_/In_2_S_3_. Some reports indicate a linear dependence of the band gap with increasing oxygen content in a In_2_S_3_ matrix which should allow direct tuning of the electronic properties of the material, but no local oxygen distribution has been reported [6]. There are different methods for the growth of In_2_O_3−*x*_S_*x*_ described in the literature, but also the unintentional incorporation in indium sulphide during processing should be considered. Processes leading to indium-based materials with mixed sulphur and oxide anions include stepwise formation of superstructures via ALD [7], sputtering techniques combined with post-growth oxidation [8], electrodeposition [9], co-precipitation sol-gel synthesis [10], or annealing of In_2_S_3_ prepared by solid-state reaction [11].

Metal alkoxides are often used as precursors in molecule-to-material processes for oxide synthesis due to preformed metal–oxygen bonds and the tunability of physical and chemical properties [12]. For instance, the formation of monomeric alkoxides of indium aminoalcoholates by the combination of sterical crowding and donor bonds from aminoligands has been described and the same strategy has proven successful for thioether-functionalised aminoalcoholates [13, 14]. The thermally labile thioether moiety in the organic backbone of thioether-functionalised aminoalcoholates allows the simultaneous incorporation of oxide and sulphide ions in the material [15]. The thioether-functionalised indium alkoxides showed promising results in hot-injection pyrolysis; however, these indium alkoxides are not volatile enough for low-pressure chemical vapour deposition.

This study describes the application of these precursors in an aerosol-assisted CVD (AACVD) process and the corresponding thin film formation and nanostructure evolution with a general composition of In_2_O_3−*x*_S_*x*_. The advantage of the AACVD approach is the independency of a precursor’s volatility because this technique relies only on the solubility of precursors and the aerosol stream is used for the transport of the precursor species to the substrate [16].

## Results and discussion

**Fig. 1:**
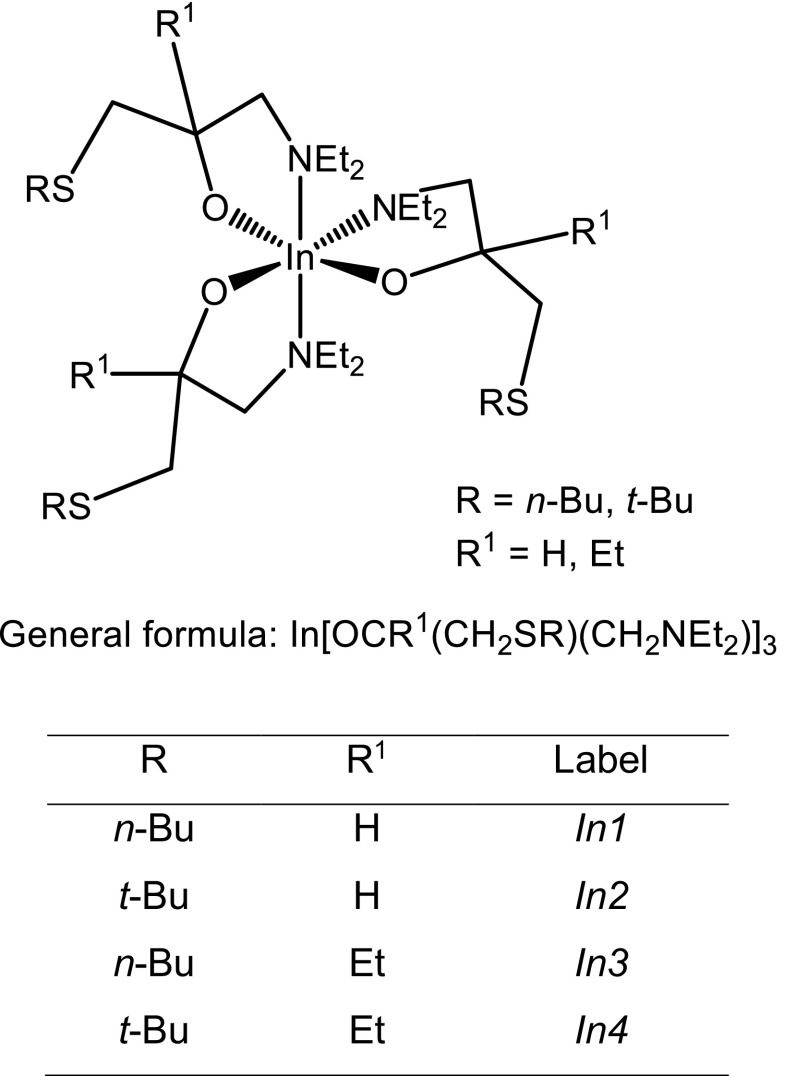
Schematic representation of the aminoalcoholate precursor species used in this study

The AACVD processes were conducted in a home-built horizontal hot-wall reactor. Process and setup details are compiled in the experimental section. Initial tests showed most consistent depositions using *n*-hexane as the solvent, which showed good solubility of the precursors, efficient aerosol generation, and fast evaporation of the solvent. The used thioether-functionalised aminoalcoholate precursors and their abbreviations are listed in Fig. 1.

**Fig. 2:**
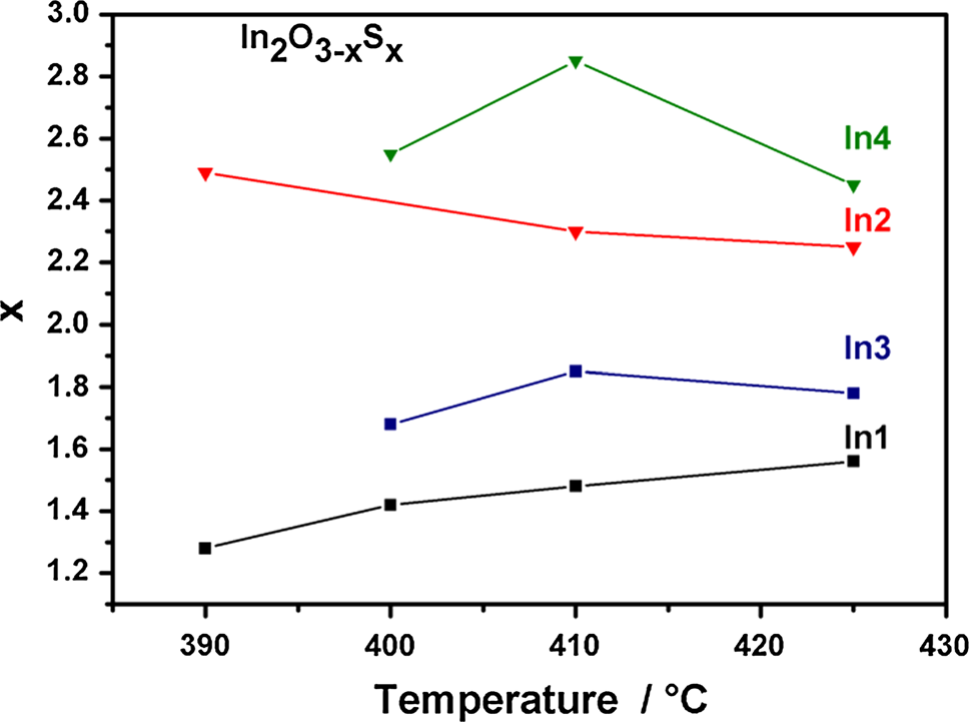
Sulphur content of In_2_O_3−*x*_S_*x*_ thin films derived from EDX spectra

We were able to convert all presented aminoalcoholates to indium oxysulphide thin film materials with varying degree of sulphur incorporation. The latter was found to be highly dependent on precursor species and deposition temperature. Exemplary AACVD experiments conducted with the gallium homologue of *In2* resulted in similar sulphur contents as reported for LPCVD coatings [15]. Figure 2 shows sulphur contents of In_2_O_3−*x*_S_*x*_ deposits determined by energy-dispersive X-ray (EDX) spectroscopy assuming that the total amount of detected sulphur is sulphidic.

The *t*-butyl thioether derivatives *In2* and *In4* show significantly higher sulphur contents compared to *n*-butyl species *In1* and *In3*. Similar behaviour has been previously observed in LPCVD of gallium homologues indicating the formation of more effective secondary decomposition products of the thioether moiety. The *n*-butyl derivatives *In1* and *In3* do not show a comparable trend. The sulphur content in the In_2_O_3−*x*_S_*x*_ deposits obtained using *In1* remains in the range of *x* = 1.28–1.56 and *In3* have slightly higher sulphur contents *x* = 1.68–1.85 (< 62% (S/(S + O))) while *In2* and *In4* deposits contain more sulphur [*x* > 2.25; >75% (S/(S + O))]. All films exhibited carbon contamination, but values are not provided due to the inaccuracy of the EDX results for elements with such a small atomic number. In general, tertiary alcoholate derivative based material shows higher carbon contamination of their secondary alcoholate counterparts. This could be caused by kinetic limitations of the heterogeneous surface reactions at the moderate decomposition temperatures in combination with rather high precursor delivery rates [17]. X-ray photoelectron spectroscopy (XPS) will give more insight into the carbon contamination, which is actually pronounced at the surface and highly diminished in the bulk of the films.

**Fig. 3:**
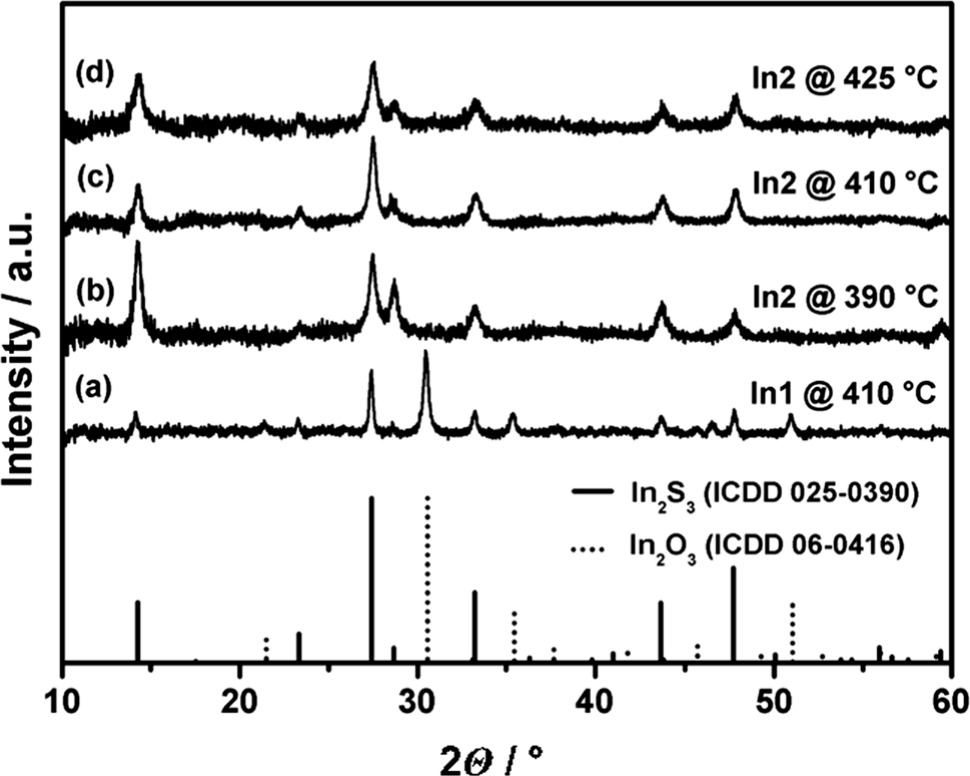
XRD patterns of thin films illustrating the formation of *a* two phases due to phase separation for low sulphur content using *In1* and *b*–*d* single phases at high sulphur content using *In2*

X-ray diffraction (XRD) of thin films obtained using *In1* and *In3* precursors indicates the presence of indium oxide and indium sulphide, which is represented by the diffractogram in Fig. 3a. In contrast, the deposits obtained from *t*-butyl thioether derivatives *In2* and *In4* show the formation of phase pure β-In_2_S_3_ in the temperature range 390–425 °C (Fig. 3b–d). Similar results showing the formation of β-In_2_S_3_ have been previously obtained for sub-micron particles obtained via hot-injection pyrolysis of thioether-functionalised indium alkoxides independent of the thioether’s alkyl chain [15]. The formation of a solid solution is strongly favoured for isostructural systems with similar ion size [18]. Despite the different crystal systems of the most stable polymorphs of In_2_O_3_ (cubic) and In_2_S_3_ (tetragonal) and oxide contents of 25% (O/(S + O)), there is no crystalline oxide phase observed in the films grown in the temperature range 390–425 °C from *In2* (Fig. 3). Although significant amounts of oxide ions should be incorporated into the parent In_2_S_3_ lattice, no shift of the reflexes in the XRD is observed. The absence of a shift in the reflections can be caused by (1) the broad peaks resulting from small crystallite size of the thin films and (2) the smaller radius of oxide ions in comparison to sulphide will not result in major physical distortion of the In_2_S_3_ host unit cell [18, 19]. However, it is also possible that the oxide content is not crystalline and the crystallisation is hampered by the sulphide; however, compared with the results on the *n*-butyl-derived materials this scenario is unlikely. Detailed studies on the role and location of the oxide ions are currently carried out and will be published elsewhere.

**Fig. 4:**
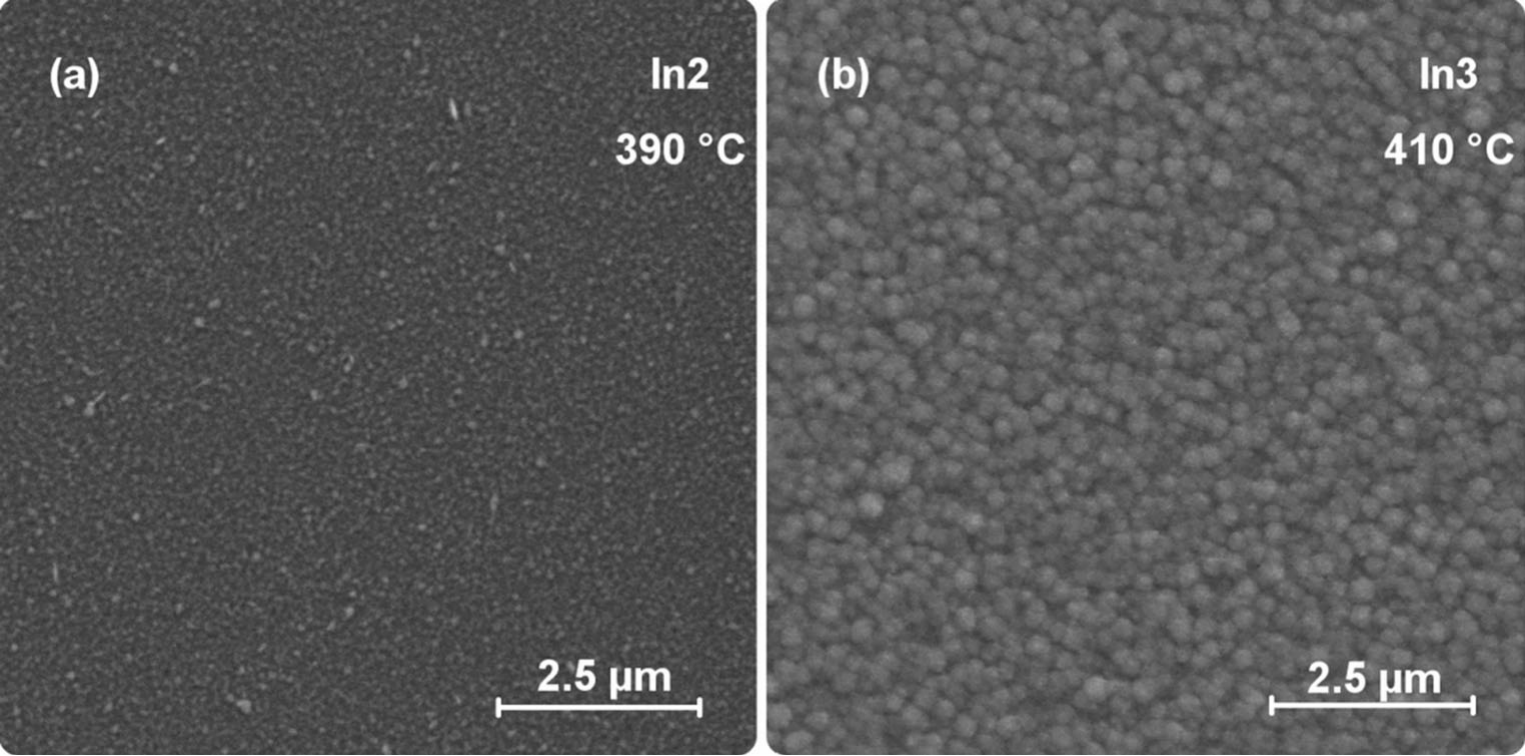
SEM micrographs of thin film samples deposited from *In2* and *In3*

Scanning electron microscopy (SEM) images of the thin films show uniform, nanostructured coatings on the Si substrates as illustrated for *In2*- and *In3*-derived films (Fig. 4). The morphology of all the thin film samples is very similar and the chosen images are representative examples.

**Fig. 5:**
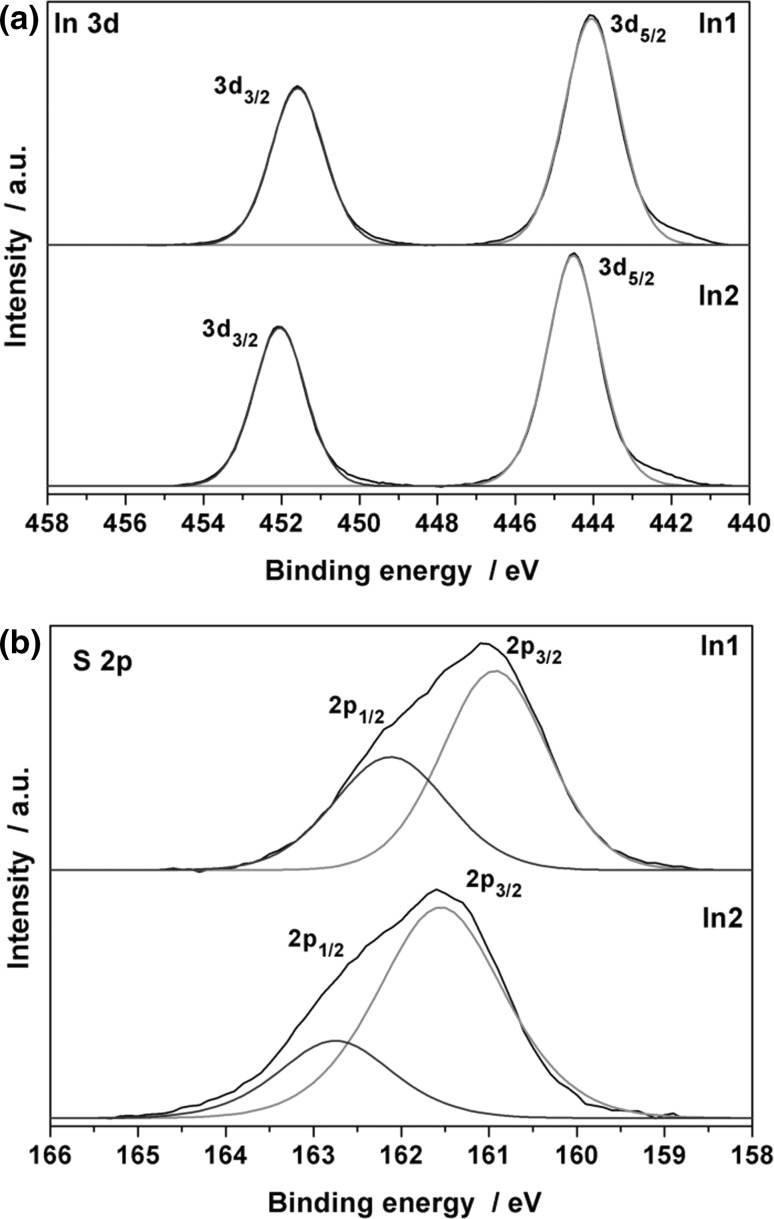
XPS spectra of the In3d and S2p region of thin films derived from *In1* (390 °C) and *In2* (410 °C)

XPS measurements were conducted to predominantly provide further information about the chemical nature of the incorporated sulphur species, as this information cannot be provided by EDX analysis. All samples have been sputtered to eliminate the well-known higher carbon content at the surface of CVD-derived coatings, which can be related mainly to the formation of carbonates and adsorbed hydrocarbons [20]. Spectral overviews before and after sputtering are shown in Fig. S1 of the Supplementary Material, which essentially show a significant decrease in the carbon signal. The carbon contamination almost vanishes after sputtering for the coatings prepared by the secondary alcoholate derivatives, while a larger fraction remains after sputtering for tertiary alcoholates such as *In4* (Fig. S1b). This is a good indication that the thermal decomposition of the tertiary alcoholate derivatives is not as clean as their counterparts derived from secondary alcohols. The carbon signal attributed to Fig. S1a is shown in higher resolution in Fig. S2 and reveals the formation of a small amount of carbonate species and a majority peak of hydrocarbons (284.2 eV). The carbon content is highly reduced after sputtering, which is also evident in the higher resolution scans. In addition, the O1s region was used to identify the oxygen species in the coatings. The parent surface contains minor fraction of water (533.5 eV), hydroxyl groups (531.5 eV), and O^2−^ (529.5 eV) [21]. Water and the majority of the hydroxyl groups are removed after sputtering, while the dominating oxygen species is oxygen bound to the metal of an oxide phase (Fig. S2b) [22]. The hydroxyl groups can be a result of water adsorption and subsequent surface reaction leading to formation of the OH-groups. The presence of a small fraction of hydroyl species after sputtering could be related to water diffusion at the grain boundaries and surface reaction of the individual grains. However, significant amounts of O^2−^ are formed during the decomposition, because alcoholates usually form oxides as long as no highly reactive secondary decomposition products emerge during the thermal decomposition and the oxide is receptive for a reaction with the secondary decomposition products [15]. Figure 5 shows the In3d and S2p regions of the spectra for sputtered samples. Only one set of signals with spin orbit splitting is found in the S2p region of the spectra and no other sulphur species could be detected. This indicates an effective and clean decomposition of the thioether moiety of the precursor species under these mild reaction conditions ≤425 °C. Furthermore, the In3d region also shows one set of symmetric signals without any shoulders for both films.

The peak positions for *In2* can be well referenced to In_2_S_3_, while signals for *In1* are shifted 0.4–0.6 eV to lower binding energies [23, 24]. This can be most likely attributed to the higher oxygen content in *In1*-derived thin films as In_2_O_3_ was reported to give signals in this range of the In3d region [25]. However, the use of a laboratory X-ray source in the XPS setup without monochromatisation should also be considered as it leads to increased signal widths and, therefore, the signal might not be sufficiently resolved to show the two different species expected for *In1*-derived coating, which showed phase separation in the XRD pattern. Sulphide contents after sputtering were found at *x* = 1.1 [37% (S/(S + O))] and 2.0 [67% (S/(S + O))], which is in good agreement with sulphide contents obtained from EDX analysis (*x* = 1.28 and 2.3 for *In1* and *In2*, respectively). However, the exact relative atomic proportion of each element in the films cannot be determined accurately by XPS because of the different etching rates of indium, sulphur, and oxygen. Therefore, we generally relate to the sulphur content determined by EDX.

**Fig. 6:**
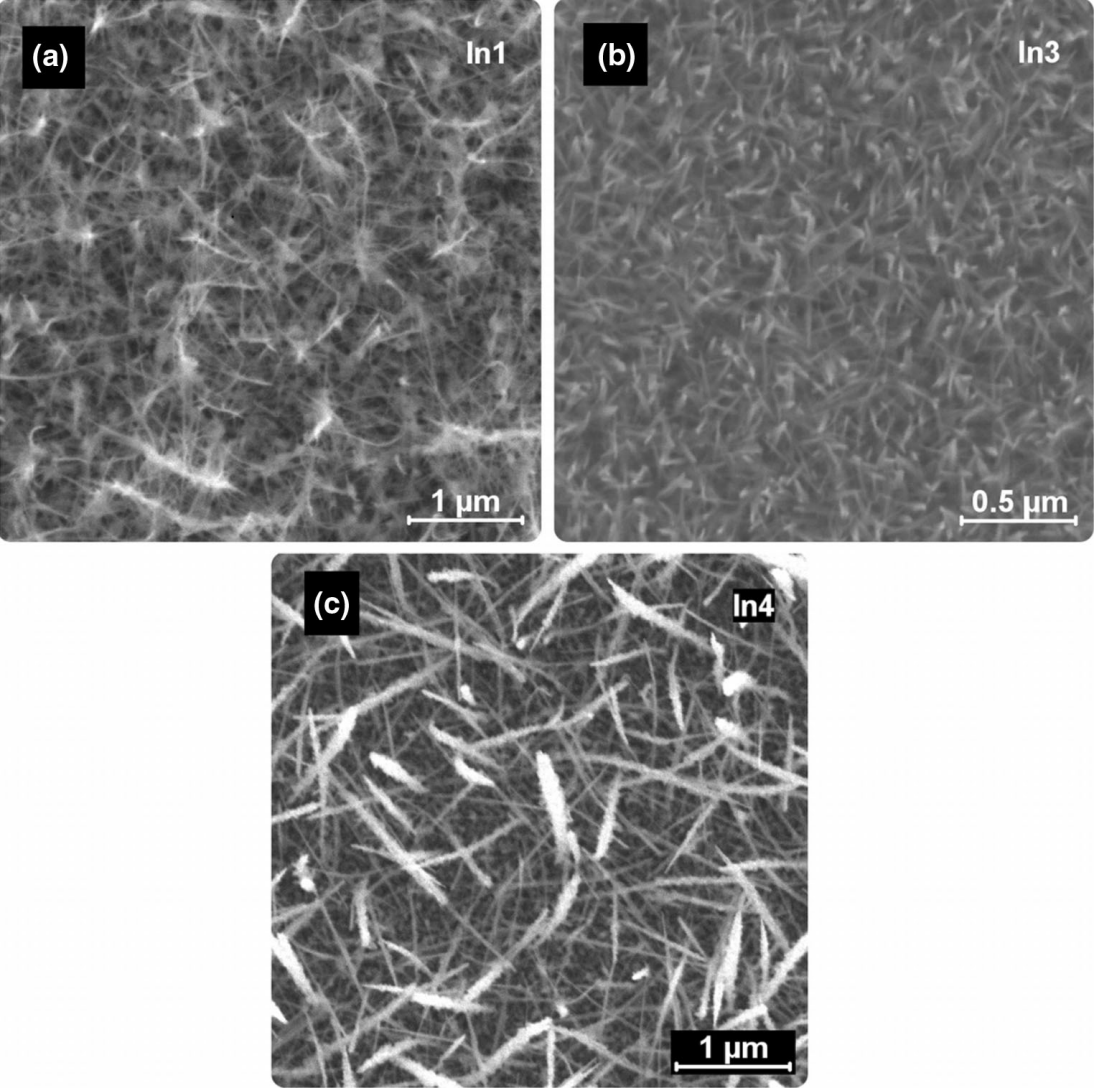
SEM micrographs of 1D structures grown from *In1*, *In3* and *In4*

At the highest applicable deposition temperature of 425 °C in combination with slightly decreased carrier gas flow rates of 40 sccm, nanowire growth has been observed for *In1*, *In3*, and *In4* (Fig. 6). The morphology and length/diameter ratio are strongly dependent on the precursor species. For *n*-butyl thioether precursor derivatives *In1* and *In3* tapered nanowires with relatively smooth surfaces were observed. On the other hand, elongated structures with significantly increased diameters as well as strong surface structuring have been observed using the *t*-butyl derivative *In4* in the AACVD process.

CVD growth of indium oxide nanowires usually requires a metal growth seed for an effective nanowire formation [26]. Similarly, the literature reports on gas phase processes mainly deal with gold-supported vapour–liquid–solid (VLS) growth [4]. No metal growth promoter is used in the here-described study and the morphology resembles the morphology of In_2_S_3_ nanostructures prepared by AACVD using a dithiocarbamate precursor without the presence of a metal growth seed [27]. However, the study on the pure elongated In_2_S_3_ nanostructures does not provide any data, which allows to assign a growth mechanism that could also be present in the here-described samples.

**Fig. 7:**
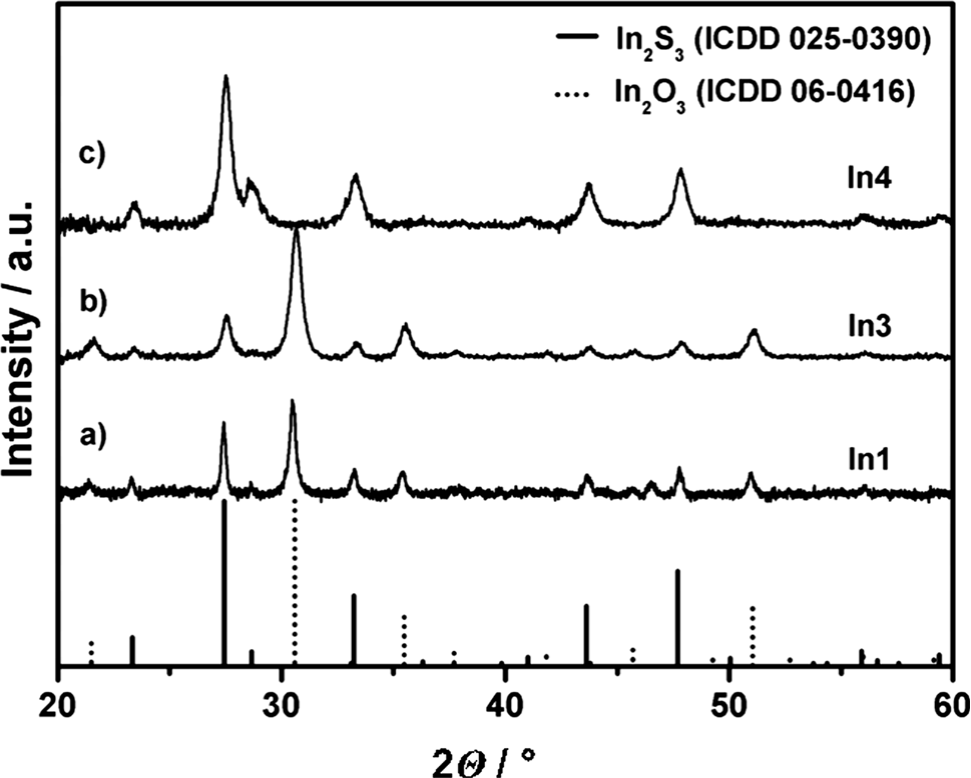
XRD patterns of 1D structures grown from thioether-functionalised indium alkoxides. *a*, *b* The presence of indium oxide and sulphide obtained by the *n*-butyl precursor derivatives *In1* and *In3*; the diffractogram in *c* shows exclusively indium sulphide as crystalline phase

Similar to the thin film samples, XRD analysis shows phase separation for the 1D structures grown from *In1* and *In3* indicated by signals from β-In_2_S_3_ and α-In_2_O_3_ (Fig. 7). This is in contrast to the phase pure In_2_S_3_ materials produced via hot-injection pyrolysis using the same precursors [15].

**Fig. 8:**
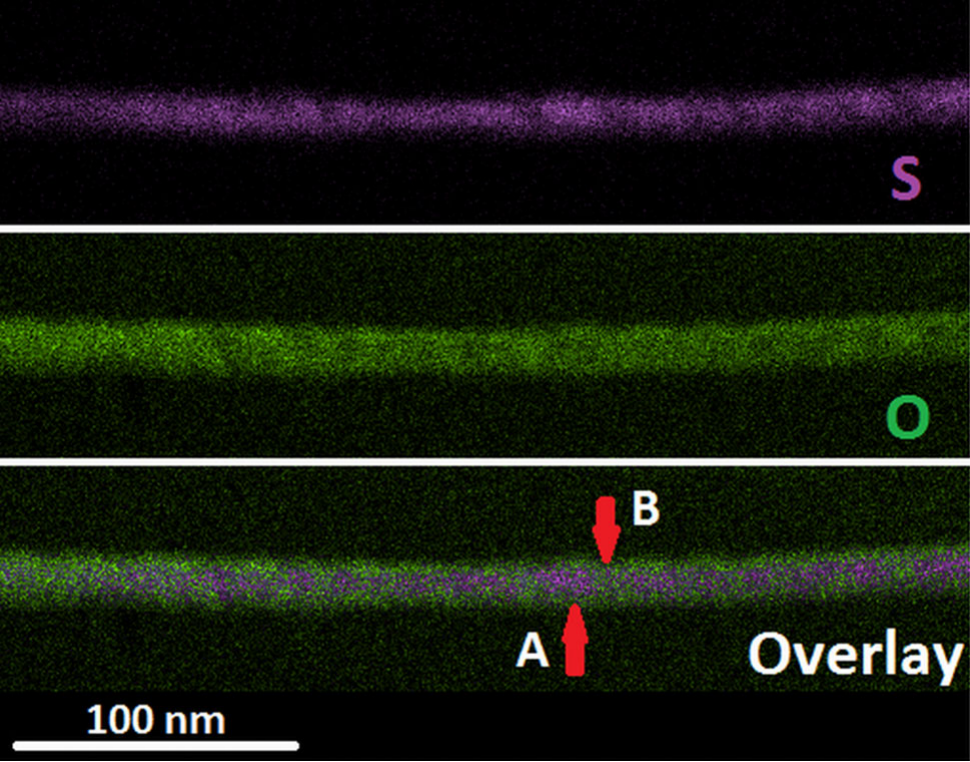
EDX mapping for sulphur and oxygen in a nanowire obtained using *In1*. The locations *A* and *B* are locations where the sulphur content was calculated. The overlay clearly shows a modulation in sulphur content in the nanowire

The most likely reason for phase separation in *In1*- and *In3*-derived nanostructures is the low sulphide content [<64% (S/(S + O)) according to EDX in Fig. 2]. Substitution of sulphur by the smaller oxygen ions will inevitably result in a collapse of the indium sulphide crystal lattice and, therefore, phase separation via spinoidal decomposition seems to be energetically favoured. For *t*-butyl thioether-functionalised precursor *In4*, sulphur contents are significantly higher according to EDX of films with approximately 83% (S/(S + O)). These lower concentrations of oxide ions most likely enable substitution in the sulphide lattice without phase separation. *In1*-derived nanowires show an inhomogeneous sulphur distribution in the nanowires with an average sulphur content of ~35% (S/(S + O)) as illustrated in the EDX mapping of an individual wire (Fig. 8). However, local concentrations vary from 23 to 56% (S/(S + O)) at different locations within the same nanowire (Fig. 8), which would explain the two phases in the XRD being present in the same nanowire. Two separate locations in close proximity to each other are illustrated in Fig. 8 and the local sulphur contents of 56% in A and 29% (S/(S + O)) in area B have been determined.

**Fig. 9:**
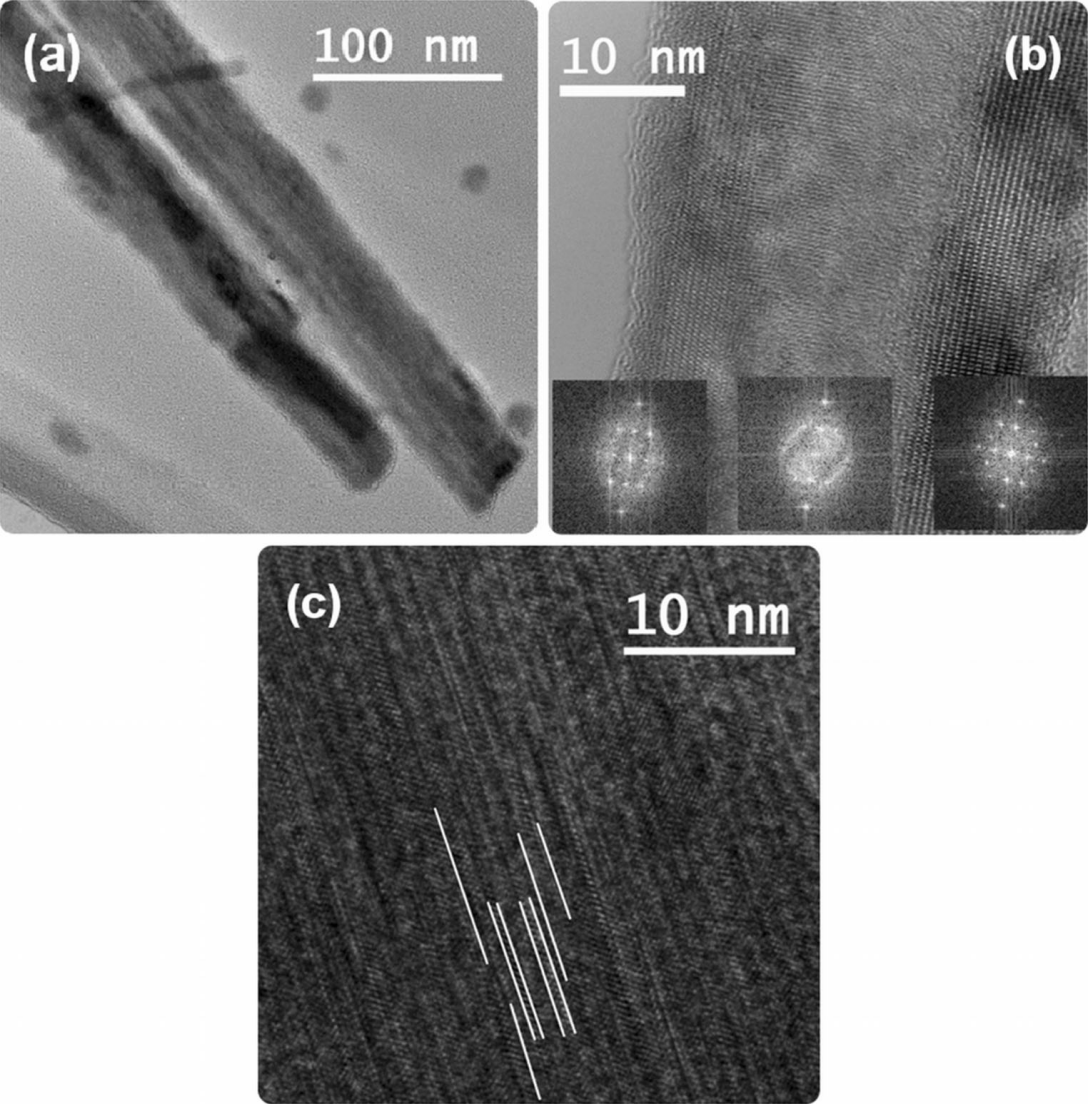
TEM micrographs of nanowires grown from *In1*. **a** Diffraction contrast pointing towards a polycrystalline wire, which is also shown in the high-resolution TEM images (**b**, **c**). The FFT patterns in **b** show a common growth axis along the nanowire with the same signal obtained for all three parts of the nanowire

Transmission electron microscopy (TEM) has been used for analysis of selected nanowires grown from aminoalcoholate *In1* to investigate the microstructure. The diffraction contrast in the TEM images already suggests a polycrystalline nature of the nanowires (Fig. 9a). High-resolution TEM micrographs, depicted in Fig. 9b, c, indicate that the nanostructure consists of a high number of crystalline domains. Images for crystallites with common interface and a common zone axis, which could have been used for an identification of the crystal structures, have not been obtained. The crystallites in Fig. 9b have different zone axes as observed from the fast Fourier transformation (FFT) pattern; however, it is evident that there is an orientation dependency as shown in the three different FFT images related to the individual grains across the nanowire. Figure 9c demonstrates that the nanowires feature a high number of stacking faults and twin domains with varying sequence and sizes and thus, very small volumes of the phases can be accommodated. Alignment of different crystal systems with common interfaces is well known for different crystal systems and is used in heteroepitaxy and heterostructure formation [28]; however, the existence in one wire is rather uncommon.

The inhomogeneity is most likely caused by spinoidal decomposition, surface diffusion of the growing material and site-specific adsorption and diffusion of decomposition products preferentially forming phase-separated indium oxide and sulphide crystallites.

## Conclusions

We have demonstrated that thioether-functionalised indium aminoalcoholates are well suited as single-source precursors for the direct synthesis of crystalline indium oxysulphide In_2_O_3−*x*_S_*x*_ thin films via AACVD processes. The resulting thin film coatings exhibit varying degrees of sulphur incorporation ranging from *x* = 1.28 to 2.80 [42–93% (S/(S + O))] depending on the selected precursor species as well as deposition temperature. Furthermore, depending on the process parameters nanowires and rod-type 1D structures are formed showing nanophase separation concomitant with high number of defects and strong twinning of the crystallites. High sulphide contents lead to materials with phase-pure sulphide crystal structure while lower sulphide content leads to phase separation and the formation of coexisting indium oxide and sulphide phases.

## Experimental

All chemicals were purchased from Sigma-Aldrich, ABCR, and TCI Europe and used as received. Manipulations of metal alkoxides were conducted taking stringent precautions against atmospheric moisture using Schlenk or modified Stock-type apparatuses. Solvents were desiccated using standard techniques and stored over Na wire, if applicable. Synthesis procedures and characterisation of the metal alkoxide precursors have been described in the literature [14]. Silicon (911) substrates were purchased from CrysTec.

### Aerosol-assisted chemical vapour deposition

AACVD was conducted in a home-built hot-wall reactor consisting of a laboratory-grade NS 29 glass tube in a tube furnace equipped with a gas inlet nozzle attached to a 50 cm^3^ flask. For a deposition, 50–75 mg of precursor were dissolved in 10 cm^3^ dry *n*-hexane and placed in the aerosol generator. The aerosol was generated ultrasonically and transported into the reactor by a stream of dry, deoxygenised nitrogen, which was controlled with a mass flow controller (MKS PR4000B) and set to 40–70 sccm. The apparatus was desiccated by heating to >400 °C for 1 h in a carrier gas stream prior to use. The Si (911) substrate temperature was initially monitored ex situ via a thermocouple under reaction conditions and found to be approx. 100 °C lower than the oven temperature. Therefore, all temperatures mentioned in the manuscript refer to the substrate temperature and not the settings of the tube furnace. In addition to thin film growth, gas phase nucleation was generally observed forming a particulate deposit at the end of the heated zone.

### Analysis techniques

Diffraction patterns were recorded on a PANanalytical XPERT Pro PW 3050/60 diffractometer with Cu Kα radiation (*λ* = 1.5406 Å) and an X’Celerator detector. Data analysis was performed using HighScore Plus software. SEM analysis was carried out with a FEI Quanta 200 FEG SEM equipped with an EDX detector for elemental analysis. TEM micrographs were acquired using a FEI TECNAI F20 TEM operated at 200 kV, which is equipped with high-angle annular dark-field (HAADF) STEM and EDX detector. XPS measurements were performed on a commercially available SPECS STM/XPS UHV setup equipped with a non-monochromatised dual-anode X-ray tube with Al and Mg Kα anodes (XRC 50, SPECS) and a hemispherical analyser PHOIBOS 100 (SPECS) with multi-channeltron detector. The base pressure was in the low 10^−9^ mbar regime. The samples were introduced into the UHV system via a load lock after mounting them on a steel plate. Samples were measured at room temperature with Al Kα radiation in the as is state and after sputtering for 45 min at 5 × 10^−6^ mbar Ar and an acceleration voltage of 1 kV. The obtained data were analysed using the commercial software CasaXPS and peak positions were corrected via the signal for graphitic carbon and double checked by measuring the Fermi Edge. Peak positions were identified using the NIST XPS database (http://srdata.nist.gov/xps/). Peaks were fitted after Shirley background subtraction using a Gaussian–Lorentian mixed peak shape (30% L) without further constraints. For comparing indium, carbon and sulphur signals the intensity was corrected by the cross sections obtained from the Elletra Trieste database (https://vuo.elettra.eu/services/elements/WebElements.html).

## Electronic supplementary material

Below is the link to the electronic supplementary material.

Supplementary material 1 (PDF 159 kb)
