# Supplementary material for: Aerosol-assisted CVD of thioether-functionalised indium aminoalkoxides
Source: Monatsh Chem. 2017 Jun 2;148(8):1385–92. doi: 10.1007/s00706-017-1980-2 (PMC5504136; doi:10.1007/s00706-017-1980-2)
Supplement: Supplementary file 1 — Supplementary material 1 (PDF 159 kb) [file 706_2017_1980_MOESM1_ESM.pdf]

Supporting Information

Aerosol assisted CVD of thioether functionalised indium aminoalkoxides

Felix Biegger, Felix Jungwirth, Michael Stanislaus Seifner, Christoph Rameshan, Sven Barth*

(a)

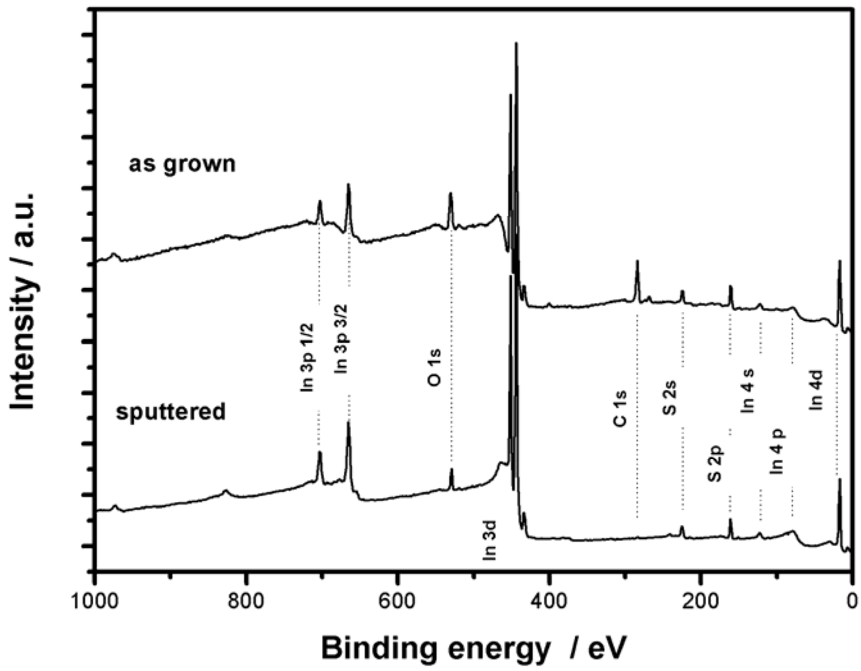

(b)

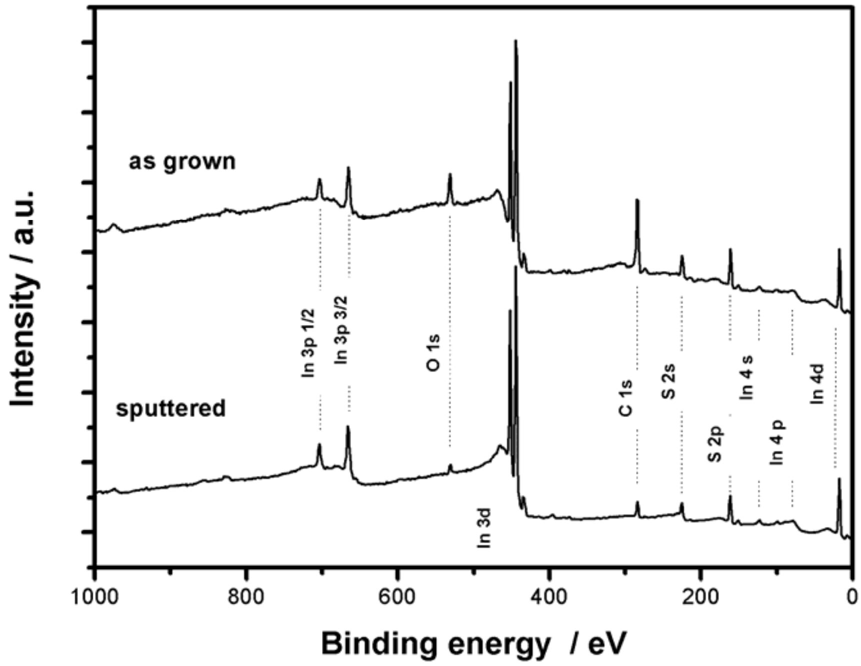

Figure S1: XPS survey of films obtained by AACVD using **In1** grown at (a) 400 °C before and after sputtering and (b) using **In4** 400 °C before and after sputtering. Spectra measured with Al Kα radiation at room temperature.

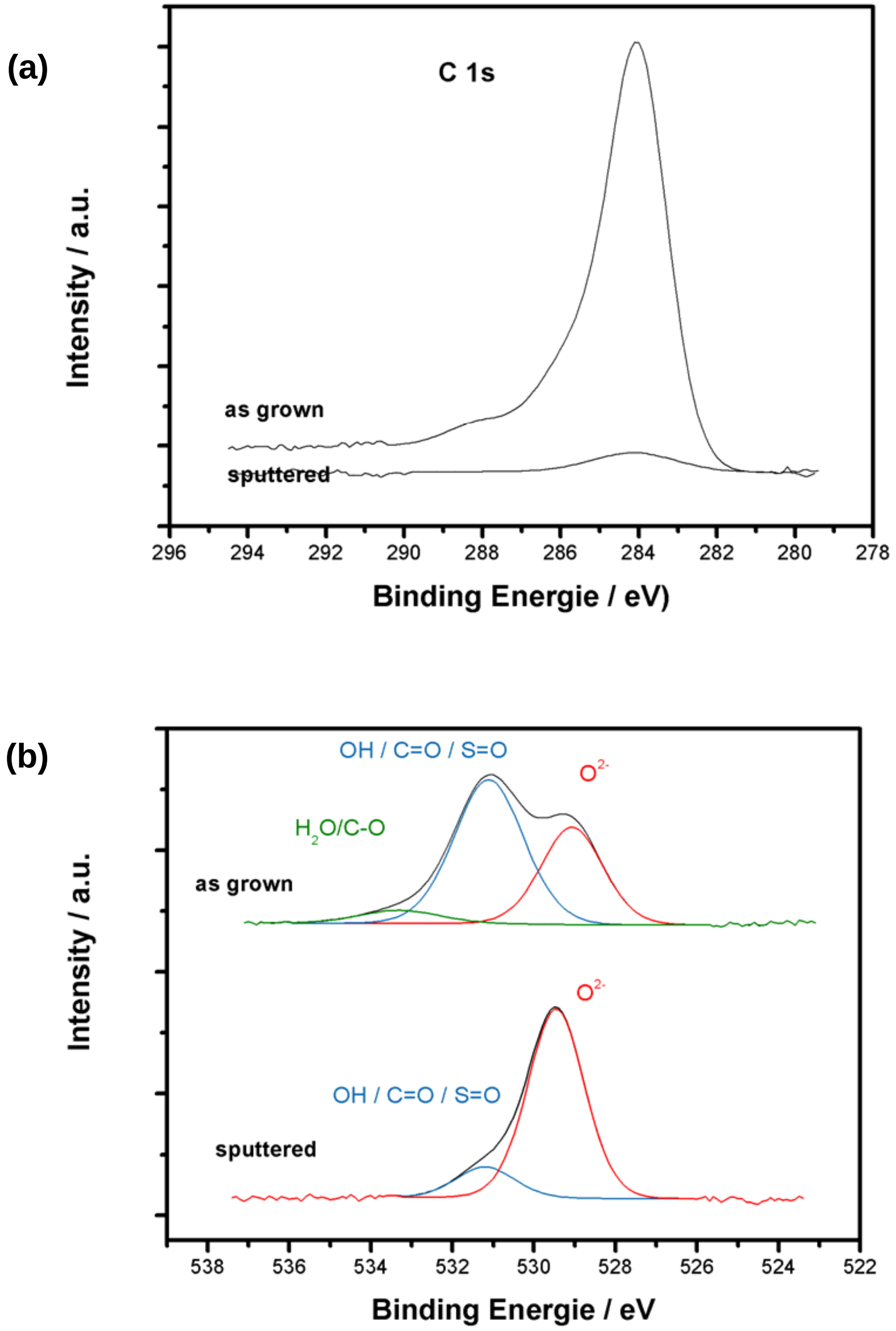

Figure S2: XPS spectra of the (a) C1s peak and (b) O1s peak of CVD deposits using **In1** at 400°C before and after sputtering recorded with an Al Kα lab source.
